# Needle electromyography abnormalities in the upper trapezius muscle in neuromuscular disorders

**DOI:** 10.55730/1300-0144.5578

**Published:** 2022-10-22

**Authors:** Halit FİDANCI, Şencan BUTURAK, İlker ÖZTÜRK, Zülfikar ARLIER

**Affiliations:** 1 Division of Clinical Neurophysiology, Department of Neurology, University of Health Sciences Adana City Training and Research Hospital, Adana, Turkey; 2 Department of Neurology, University of Health Sciences Adana City Training and Research Hospital, Adana, Turkey

**Keywords:** Electromyography, fasciculation potentials, spinal accessory nerve, trapezius muscle

## Abstract

**Background/aim:**

Needle electromyography (EMG) abnormalities in the trapezius muscle (TM) can be seen in neuromuscular disorders. The aim was to determine the characteristics of needle EMG abnormalities observed in the TM in neuromuscular disorders.

**Materials and methods:**

The data of patients who applied to the Clinical Neurophysiology Laboratory of University of Health Sciences Adana City Training and Research Hospital between December 2018 and October 2021 were reviewed. Polio survivors, amyotrophic lateral sclerosis (ALS) patients, patients with sensorimotor polyneuropathy, patients with spinal cord lesions involving C2/C3/C4 segments, patients with spinal accessory nerve (SAN) lesions, neuralgic amyotrophy (NA) patients, and patients with myopathy were included. Needle EMG findings of the upper TM of the patients were analyzed. Positive sharp waves, fibrillation potentials, fasciculation potentials, myotonic discharges, and motor unit action potential (MUAP) changes were considered needle EMG abnormalities.

**Results:**

Eighty-one polio survivors, 23 ALS patients, 39 patients with sensorimotor polyneuropathy, 10 patients with cervical spinal lesions, eight NA patients, seven patients with SAN lesions, and three patients with myopathy were included in the study. Fifteen (65.2%) ALS patients, 18 (22.2%) polio survivors, three (30%) patients with cervical spinal lesions, two (5.1%) patients with sensorimotor neuropathy, one (12.5%) NA patient, seven (100%) patients with SAN lesions, and two (66.7%) patients with myopathies had at least one needle EMG abnormality in the TM. Fasciculation potentials in the TM were seen in 10 (43.5%) ALS patients. In four patients with SAN lesions and one polio survivor, MUAP could not be obtained from the TM.

**Conclusion:**

There may be more frequent needle EMG abnormalities, particularly in ALS patients and patients with SAN lesions. Since the number of patients with myopathy included in this study was low, it is difficult to comment on the needle EMG features of the TM for these patients. In addition, this study indicated that fasciculation potentials in the TM are typical in ALS patients and that MUAP may not be obtained from the TM in patients with SAN lesions.

## 1. Introduction

Although the motor innervation of the trapezius muscle (TM) is still controversial, it is traditionally known that the spinal accessory nerve (SAN) originating from the upper spinal segments provides motor innervation of the TM [1–3]. Nerve conduction studies and needle electromyography (EMG) play an important role in diagnosing disorders that may cause weakness or atrophy in TM. Examination of the TM with needle EMG may provide important information regarding the physiology of the motor neuron or axons of the SAN or the presence of a myopathy that may cause the TM abnormality. In addition, with needle EMG and nerve conduction studies, differential diagnosis can be made, or important clues can be obtained for prognosis. The needle EMG abnormalities such as neurogenic motor unit action potentials (MUAPs) or active denervation findings in the TM can be found in SAN lesions or neuralgic amyotrophy (NA), or disorders affecting motor neurons in the cervical spinal cord such as amyotrophic lateral sclerosis (ALS) [4–10]. Moreover, the TM may be directly affected in conditions such as myopathy, and in this case, MUAPs with myopathic features may develop in the TM [8]. In this study, the aim was to determine the characteristics of needle EMG findings that can be seen in TM in neuromuscular diseases.

## 2. Materials and methods

### 2.1. Subjects

Patients over the age of 18 years, who applied to our clinical neurophysiology laboratory between December 2018 and October 2021, and whose upper TM was examined by needle EMG, were included in this retrospective study. Ethics approval was obtained from the local ethics committee of our hospital (number: 98/1748,2022). To be included, patients were required to have one of the following diseases or conditions and the TM of these patients should have been examined using needle EMG: 1) History of poliomyelitis and neurological deficit due to poliomyelitis; 2) ALS; 3) Sensorimotor polyneuropathy; 4) Spinal cord lesion involving C2/C3/C4 segments; 5) NA; 6) SAN lesion; or 7) Myopathy. All patients had brain and cervical magnetic resonance imaging (MRI). Patients with lesions in the brain stem were excluded from the study. Patients with more than one of these diseases or neurodegenerative diseases were not included in the study. A total of 171 patients were included. If the complaints were bilateral in patients with a cervical spinal lesion or if the affected upper extremity of polio survivors was bilateral, both TMs were examined by needle EMG. Therefore, needle EMG was performed on both TMs in one polio survivor and three patients with cervical spinal lesions. A total of 175 TMs of 171 patients (right TM in 145 patients, left TM in 22 patients, both TMs in 4 patients) were examined using needle EMG.

### 2.2. Nerve conduction studies and needle EMG

Electrodiagnostic tests were performed using the Cadwell Sierra Summit EMG unit (Cadwell Laboratories, Kennewick, Washington, USA). Nerve conduction studies were performed if the extremity temperature was above 32°C. Cold extremities were warmed. High pass and low pass filters for motor and sensory nerve conduction studies were set to 20 Hz-10kHz and 20 Hz-2kHz, respectively. Stimulation and recording were performed with surface electrodes. Routine nerve conduction studies were performed with previously suggested methods [11–13]. For reference values of nerve conduction studies, reference values from previous studies were used [12,13]. Nerve conduction study of the SAN was performed with surface electrodes using the previously suggested method [8,9].

Concentric needle electrodes (length = 50 mm, diameter = 0.46 mm, Bionen Medical Devices, Florence, Italy) were used for needle EMG examination. Needle EMG was applied to the upper TM and performed visually. Positive sharp waves (PSWs) and fibrillation potentials (FPs), fasciculation potentials, myotonic discharges, and complex repetitive discharges (CRDs) during rest were carefully analyzed. At least ten to twenty MUAPs were analyzed during mild contraction. A MUAP was considered neurogenic if its duration and amplitude were >15 ms and >3.5 mV, respectively. If the MUAP duration was <5 ms with an amplitude of <0.15 mV, these MUAPs were considered myopathic. The interference pattern was also included in the analyses. If the recruitment frequency was >10 Hz, it was considered reduced. PSWs, FPs, fasciculation potentials, myotonic discharges, reduced recruitment, and MUAP abnormalities were considered needle EMG abnormalities.

### 2.3. Polio survivors and ALS patients

Polio survivors typically have a history of poliomyelitis and weakness/muscle atrophy in muscles of at least one extremity. Patients with the postpolio syndrome were identified as previously suggested [14]. In polio survivors, if needle EMG abnormality was found in two muscles with different nerve and segment innervation in the upper or lower extremity, the cervical or lumbosacral region was considered to be affected. Patients diagnosed with definite or probable ALS according to Awaji diagnostic criteria were included in the study [15].

### 2.4. Patients with sensorimotor polyneuropathy

Considering the previously suggested electrodiagnostic features of polyneuropathy, patients with abnormalities in motor and sensory nerve conduction studies of both sural and posterior tibial/peroneal nerves were considered to have sensorimotor polyneuropathy [16,17]. The presence of demyelinating polyneuropathy was determined according to previously determined criteria [17,18]. In addition, patients typically have paresthesias or abnormalities in sensory examination or reduced/loss of deep tendon reflexes [16,17].

### 2.5. Patients with a spinal lesion in C2/C3/C4 segments

Individuals with MRI findings compatible with spinal cord lesions including C2/C3/C4 segments were included in the study.

### 2.6. Patients with NA

Patients with NA were required to have the following: 1) The complaints started with severe pain and the complaints decreased or completely disappeared within days or weeks; 2) Muscle weakness and atrophy developed after the decrease or disappearance of the pain [10,19].

### 2.7. Patients with SAN lesions

Patients with SAN lesions were required to have had weakness of the TM associated with trauma or neck dissection or a mass in the neck. SAN compound muscle action potential (CMAP) was considered abnormal if the SAN CMAP amplitude was reduced by more than 50% compared to the contralateral SAN CMAP amplitude.

### 2.8. Patients with myopathy

Genetic test or needle EMG findings or muscle biopsy of the patients compatible with myopathy were included in the study.

### 2.9. Statistical analysis

Categorical variables were summarized as percentages and frequencies. The Pearson’s chi-squared and Fisher’s exact tests were used to analyze categorical variables. P < 0.05 was considered to be statistically significant. The statistical package for the social sciences (SPSS IBM Corp; Armonk, NY, USA) 22.0 was used to perform the statistical analysis.

## 3. Results

**Figure 1:**
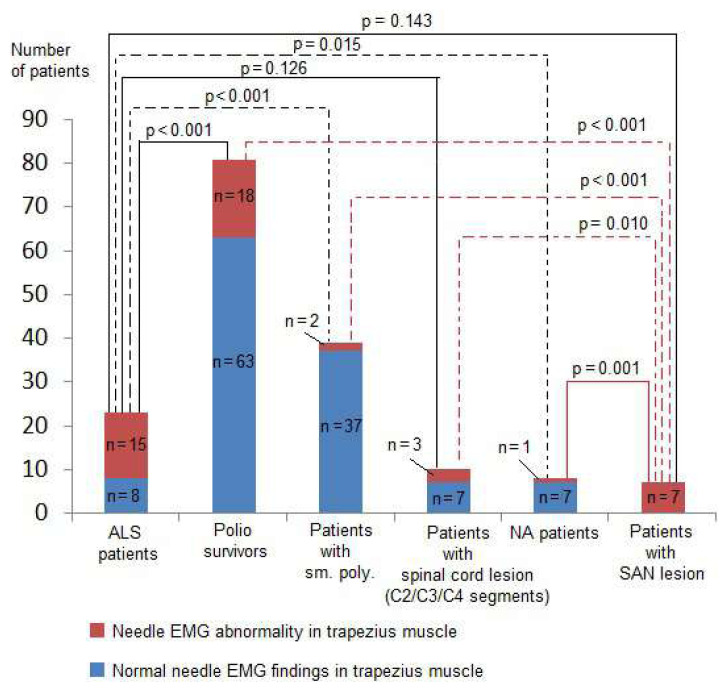
Comparison of the number of patients with at least one needle EMG abnormality in the trapezius muscle between patient groups. ALS: amyotrophic lateral sclerosis, EMG: electromyography, NA: neuralgic amyotrophy, SAN: spinal accessory nerve, sm. poly.: sensorimotor polyneuropathy.

Two patients were excluded from the study due to a history of both poliomyelitis and polyneuropathy. In addition, one ALS patient had polyneuropathy and was also excluded. Eighty-one polio survivors, 23 ALS patients, 39 patients with sensorimotor polyneuropathy, 10 patients with cervical spinal lesions, 8 patients with NA, 7 patients with SAN lesions, and 3 patients with myopathy were included in the study. Table 1 indicates the needle EMG findings of the TM among the patient groups. A comparison of the number of patients with at least one needle EMG abnormality in the TM among patient groups is shown in Figure 1.

### 3.1. Polio survivors

Fifty-six (69.1%) of the patients were male. The mean age of the patients was 49.6 ± 9.3 (min-max 31–79) years. The right TMs of 73 patients, the left TM of 7 patients, and the bilateral TMs of one patient were analyzed by needle EMG. The number of patients with weakness in one lower extremity, both lower extremities, one upper extremity, one upper and one lower extremity, bilateral lower extremities and one upper extremity, and all extremities were 31, 40, 1, 2, 6, and 1, respectively. According to the needle EMG findings, the lumbosacral region was affected without needle EMG abnormality of the TM in 45 patients, and the lumbosacral and cervical regions were affected without needle EMG abnormality of the TM in 18 patients. In addition to affecting the cervical and lumbosacral regions of the other 18 patients, needle EMG abnormalities were also found in the TM. There were 18 patients with postpolio syndrome. Two of the patients with postpolio syndrome and 16 of the other 63 patients had needle EMG abnormality in the TM; however, this difference was not significant (p = 0.335). Needle EMG findings of muscles in polio survivors are shown in Table 2. Fasciculation was observed in the tibialis anterior muscle of one of the patients.

### 3.2. ALS patients

The mean age of 23 ALS patients (13 male, 10 female) was 56.2 ± 11.6 (30–71) years. The time interval between the onset of the complaints and the timing of the electrodiagnostic test was 12.6 ± 6.5 (min-max 3–24) months. The right TMs in 20 patients and the left TMs in 3 patients were examined using needle EMG. The diagnosis of ALS was definite in 15 patients and probable in 8 patients. Needle EMG abnormality of the TM was found in 11 and 4 of the patients with definite and probable diagnoses of ALS, respectively (p = 0.371). The region of ALS onset was the upper extremity in 15 patients, the lower extremity in 4 patients, and the bulbar region in 4 patients. Three of the bulbar-onset ALS patients, 10 of the upper-extremity-onset ALS patients, and two of the lower-extremity-onset ALS patients had needle EMG abnormalities in the TM. Ten patients (43.5%) had both neurogenic MUAPs and PSWs or FPs or fasciculation potentials in the TM. Needle EMG was applied to the tongue in 12 patients, but five patients (41.7%) could not relax. The TM was examined with needle EMG during rest in all patients. Table 3 shows the needle EMG findings of ALS patients.

### 3.3. Patients with sensorimotor polyneuropathy

The mean age of the patients with sensorimotor polyneuropathy was 57.1 ± 13.4 (min-max 18–78) years. Thirty-five of the patients were male. The right TMs were examined in 35 patients, and the left in four patients. Thirty-two of 39 patients had diabetes mellitus. The clinical and electrophysiological features of 5 patients were compatible with Chronic Inflammatory Demyelinating Polyneuropathy (CIDP). There were two patients with hereditary sensorimotor polyneuropathy whose genetic tests were not available but similar complaints had been seen in family members. One CIDP patient and one hereditary sensorimotor neuropathy patient had high amplitude and long duration MUAPs in the TM.

### 3.4. Patients with a spinal lesion in C2/C3/C4 segments

**Figure 2:**
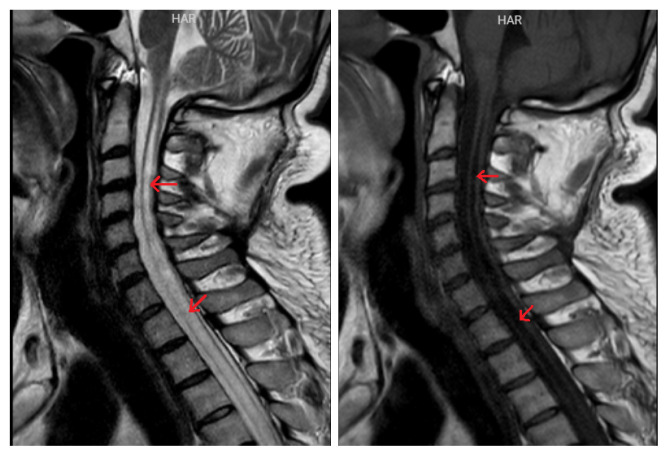
Magnetic resonance imaging of the patient with syringomyelia in the cervical region. Red arrows: Cervical spinal cord syrinx.

**Figure 3:**
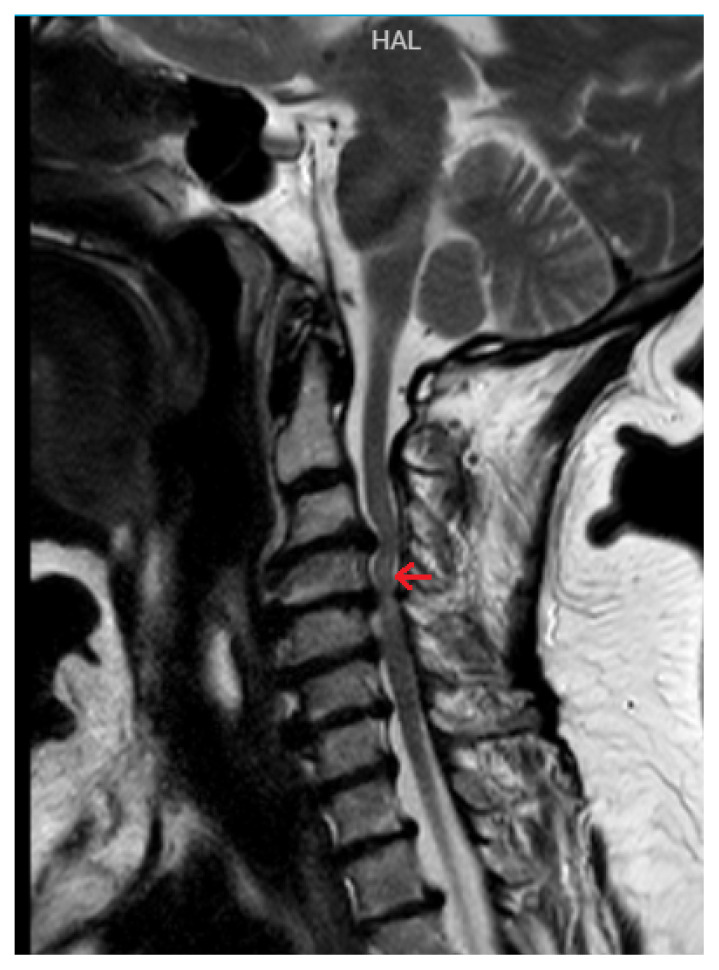
Magnetic resonance imaging of the patient with cervical spine myelomalacia due to disc herniation. Red arrow: Cervical spinal cord myelomalacia.

The mean age of the patients (8 male, 2 female) was 56.0 ± 24.7 (min-max 31–80) years. The right TM was examined in five patients, both TMs in three patients, and the left TM in two patients. Nine patients had cervical myelopathy including C2/C3/C4 segments due to cervical disc herniation. One patient had cervical syringomyelia (Figure 2). The patient with syringomyelia had high amplitude and long duration MUAPs in both TMs. In addition, this patient had PSWs/FPs and neurogenic MUAPs in both first dorsal interosseous muscles and right biceps brachii, deltoid, and triceps muscles. One patient with cervical disc herniation had PSWs, FPs, and CRDs in the bilateral TMs, as well as neurogenic MUAPs in both muscles. Another cervical radiculopathy patient had CRDs with PSWs/FPs in the right TM. Figure 3 shows the MRI of this patient.

### 3.5. NA patients

The mean age of eight NA patients (8 male, 2 female) was 37.9 ± 15.6 (min-max 19–62) years. The time interval between the onset of complaints and the timing of the electrodiagnostic tests was between 2 months and 4 months in all patients. All patients had weakness in only one upper extremity (right extremity in five patients, left extremity in three patients). In patients with normal needle EMG of the TM, the needle EMG findings were as follows: abnormalities in the serratus anterior muscle and muscles innervated by the C5, C6 segments in four patients; abnormality in C7, C8, T1 innervated muscles and serratus anterior muscle in one patient; and two patients had abnormalities only in muscles innervated by C5, C6, C7 segments. In one NA patient, in addition to finding needle EMG abnormality in the TM, and the supraspinatus and deltoid muscles were also abnormal.

### 3.6. Patients with SAN lesion

**Figure 4:**
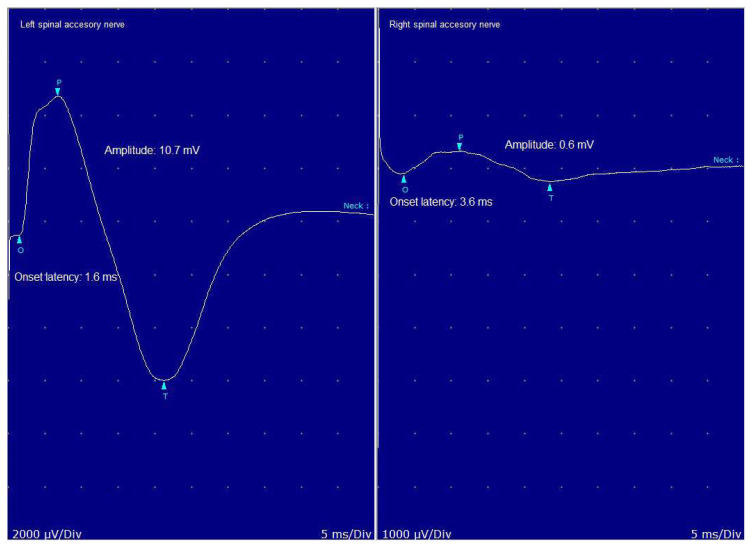
Nerve conduction study of a patient with spinal accessory nerve lesion.

The clinical and electrodiagnostic features of the patients with SAN lesions are shown in Table 4. Figure 4 shows the SAN nerve conduction study of Patient 3 with a SAN lesion.

### 3.7. Patients with myopathy

There were three male patients aged 42, 45, and 66 years with myopathy. In these patients, the right TMs were examined with needle EMG. The 66-year-old patient with myopathy had a diagnosis of myotonic dystrophy, which was also confirmed by genetic testing, and had myotonic discharges and myopathic MUAPs in other muscles, including the right TM. Genetic tests for the other patients were not available, but similar complaints were seen with in their families, and the diagnoses of these two patients were compatible with limb-girdle muscular dystrophy and distal myopathy. The patient with limb-girdle muscular dystrophy had PSWs/FPs in the right biceps brachii, vastus lateralis muscles, and the TM. Myopathic MUAPs were found in the biceps brachii and vastus lateralis muscles.

## 4. Discussion

Although needle EMG abnormalities of the TM were observed in each of the diseases included in this study, they were more common in ALS patients and patients with SAN lesions. However, since there were only three patients with myopathy, it should be kept in mind that this finding may not be valid for myopathy.

The coexistence of PSWs, FPs, fasciculation potentials, and neurogenic MUAPs in a muscle is important for the diagnosis of ALS [15]. Sonoo et al. reported that both active denervation and MUAP changes in the TM according to the Awaji criteria were found in 45% of ALS patients [6]. Similarly, the rate found in the present study was 43.5%. Again, in research by Sonoo et al., MUAP changes, fasciculation potentials, and PSWs/FPs were seen in 64%, 39%, and 45% of patients, respectively [6]. Although the frequencies of MUAP changes and fasciculation potentials were close to those found by Sonoo et al., a higher rate of PSWs/FPs in the TM was seen in the present study. This can be explained by the high rate of patients with definite ALS in the present study according to the Awaji criteria [15]. These findings and the problem of relaxation during the examination of the tongue with needle EMG increase the importance of the evaluation of the TM with needle EMG in ALS [6,20,21]. Previous studies have indicated that active denervation was observed more frequently in distal muscles such as the first dorsal interosseous, abductor pollicis brevis, and tibialis anterior in ALS [4,22]. Similar results were partially obtained in the present study. Pathological studies found that motor axons degenerate from distal to proximal [23]. This may mean that innervation may be lost earlier in distal extremity muscles which have long axons in the early stages of ALS [22]. However, it is contrary to the fact that denervation can also be seen in the TM, which has a shorter axon, in the early stages of ALS.

The absence of PSWs/FPs in the TMs of polio survivors and the presence of neurogenic MUAPs in 20% of the patients indicated that the TM was less affected in polio survivors than in ALS patients. In addition, in the present study, the TM had the least needle EMG abnormality compared to other muscles in polio survivors. An interesting finding regarding polio survivors was that needle EMG abnormality of the TM did not occur alone and was observed in addition to the involvement of both cervical and lumbosacral regions. Although the motor innervation of the TM is a controversial issue, considering that the lower medulla, as well as the upper cervical spinal segment, may have some role in the innervation of the TM [5,6], it can be thought that there is rarely permanent damage to the upper cervical spinal segment or bulbar region in polio survivors. However, as shown in the present study, when there is widespread involvement, that is, if there is involvement in the cervical and lumbosacral regions, these segments may be affected. Lastly, the fact that needle EMG abnormality of the TM was not different between patients with and without postpolio syndrome may support that needle EMG findings of the TM may not be distinctive in both groups [24].

Fasciculation potentials are not specific to ALS and can occur in many conditions [25–27]. In the present study, fasciculation in the TM was seen only in ALS patients, with fasciculation potentials found in the tibialis anterior muscle of one polio patient. These findings indicate that the presence of fasciculation potentials in the TM is an important finding for the diagnosis of ALS [6,20,21].

The timing of electrodiagnostic tests affects the findings of needle EMG and nerve conduction studies. In patients with SAN lesions, only one patient did not have PSW/FP in the TM; however, the electrodiagnostic test for this patient was performed two years after neck dissection. Remmler et al. and Tsuji et al. also found PSWs/FPs in more than 50% of SAN lesions associated with neck surgery [7,28]. In addition, since the SAN lesion is a peripheral nerve lesion, it can be seen as complete paralysis, therefore, it is thought that the number of patients in whom MUAP in the TM or SAN CMAP could not be obtained was high in the current research. In Laughlin et al., the CMAP amplitude was between 0 and 1 mV in most of the patients in electrodiagnostic tests performed three months after neck dissection [9]. In addition, MUAP could not be achieved after 3 months in 11 of 16 patients in that same study. Unlike these studies, all of the patients included in the current study did not have a SAN lesion that developed solely after neck dissection; however, this study, in addition to those previously mentioned, demonstrates that PSW/FPs are frequent in the acute period of SAN lesions and that the absence of MUAPs or reduced SAN CMAP amplitude is an important finding for SAN lesions [7,9,28]. In addition to patients with SAN lesions, it should be kept in mind that MUAP could not be obtained in the TM of only one polio survivor.

The SAN runs upwards in the spinal canal [29], so if only radiculopathy is present, the TM is not affected, and therefore weakness of the TM is not expected. However, needle EMG abnormalities may develop in the TM when motor neurons of SAN in the spinal cord are affected. Sonoo et al. found MUAP changes in the TM in approximately 20% of patients with cervical spondylosis, but without PSWs/FPs [6]. The present study included patients with spinal cord involvement. PSWs/FPs and CRDs were observed in two patients. These two patients had cervical myelopathy due to radiculopathy. Other patients also had cervical myelopathy, but there was no needle EMG abnormality of the TM in these patients. Although it is difficult to explain this situation, these findings can likely be explained by the timing of the electrodiagnostic tests or the severity of the damage to the cervical spinal motor neurons. Other possible causes may be the effect of intact spinal segments other than the affected spinal segment on the TM, or the contribution of the lower medulla to the innervation of the TM, although it is controversial [6,30]. One patient had cervical syringomyelia and neurogenic MUAPs in both TMs. In research by Veilleux and Stevens, needle EMG abnormality was found in 75% of patients with syringomyelia [31]. However, the TM was not among the muscles examined by needle EMG in their study, and there were fewer needle EMG abnormalities in the proximal muscles of the upper extremity than in the hand muscles. Therefore, the needle EMG abnormality of the TM found in the present study is important as it indicates that motor neurons of the upper cervical region may also be affected in syringomyelia.

Needle EMG abnormalities have been found in many muscles, including the TM, in NA patients [10,19]. In this study, needle EMG abnormality of the TM was found in only one of eight patients. Although this number seems low, it should be kept in mind that the TM is not examined with needle EMG in every patient diagnosed with NA in the neurophysiology laboratory used in this research.

The majority of polyneuropathy patients included in the present study had diabetes mellitus. Diabetes mellitus is known to cause length-dependent polyneuropathy. Therefore, neurogenic MUAPs may be present in distal extremity muscles, whereas needle EMG is expected to be normal in proximal extremity muscles [32,33]. Consistent with this situation, the needle EMG of the TM was normal in patients with polyneuropathy due to diabetes mellitus in the current study. Only one patient with CIDP and one patient with hereditary polyneuropathy had neurogenic MUAPs in the TM. These needle EMG abnormalities may be due to severe axonal degeneration or the absence of length-dependent polyneuropathy in these patients.

Only three myopathy patients were included in the current study. Two of these patients had needle EMG abnormality of the TM. It is known that the muscles are affected according to the type of myopathy. For example, in some myopathies, the proximal muscles are predominantly affected, or in facioscapulohumeral muscular dystrophy, the facial and scapular muscles are frequently affected, or in distal myopathies, the distal muscles are prominently affected [34,35]. As expected, in the present study, myopathic MUAPs and PSWs and FPs or myotonic discharges were observed in the TM of patients with myotonic dystrophy and limb-girdle muscular dystrophy, whereas needle EMG of the TM was normal in the patient with distal myopathy.

In addition to being a retrospective study, this study had several limitations. First, the number of patients was low in some patient groups. Second, there was insufficient data on the timing of electrodiagnostic tests in most of the patients, except in ALS patients, NA patients, and patients with SAN lesions. Third, one patient with myopathy and one patient with hereditary polyneuropathy had genetic testing. The diagnoses of other hereditary polyneuropathy and myopathy patients were made using clinical and electrodiagnostic tests. It may be important to examine the TM with needle EMG in further studies that include genetic tests. Finally, examination of the bilateral TM with needle EMG in a small number of patients can be considered a limitation.

In conclusion, although this study demonstrated that needle EMG abnormality of the TM is present in many neuromuscular diseases, it also pointed out that ALS patients and patients with SAN lesions have a greater number of needle EMG abnormalities in the TM. However, it should be kept in mind that due to the small number of myopathy patients in this study, it would be difficult to interpret needle EMG abnormality in myopathy patients. In addition, it can be concluded that fasciculation potentials and absence of MUAP in the TM have an important role in the diagnosis of ALS and SAN, respectively.

## Figures and Tables

**Table 1:** Needle EMG findings of trapezius muscle among the patient groups.

Needle EMG findings	Number of patients (%)						
	Polio survivors (n = 81)	ALS patients (n = 23)	Patients with sensorimotor poly. (n = 39)	Patients with C2/C3/C4 spinal les. (n = 10)	NA patients (n = 8)	Patients with SAN les. (n = 7)	Patients with myopathy (n = 3)
PSW / FP	0 (0)	14 (60.9)	0 (0)	2 (20)	1 (12.5)	6 (85.7)	1 (33.3)
Fasciculation	0 (0)	10 (43.5)	0 (0)	0 (0)	0 (0)	0 (0)	0 (0)
CRD	0 (0)	0 (0)	0 (0)	2 (20)	0 (0)	0 (0)	0 (0)
Myotonic discharges	0 (0)	0 (0)	0 (0)	0 (0)	0 (0)	0 (0)	1 (33.3)
MUAP amp.↑ dur.↑	17 (21.0)	10 (43.5)	2 (5.1)	2 (20)	1 (12.5)	2 (28.6)	0 (0)
MUAP amp.↓ dur. ↑	0 (0)	0 (0)	0 (0)	0 (0)	0 (0)	0 (0)	1 (33.3)
Recruitment ↓	17 (21.0)	15 (65.2)	2 (5.1)	2 (20)	1 (12.5)	3 (42.9)	0 (0)
Absence of MUAP	1 (1.2)	0 (0)	0 (0)	0 (0)	0 (0)	4 (57.1)	0 (0)
At least one abnormality	18 (22.2)	15 (65.2)	2 (5.1)	3 (30)	1 (12.5)	7 (100)	2 (66.7)

ALS: amyotrophic lateral sclerosis, amp.: amplitude, CRD: complex repetitive discharge, dur.: duration, EMG: electromyography, FP: fibrillation potential, les: lesion, MUAP: motor unit action potential, NA: neuralgic amyotrophy, poly: polyneuropathy, PSW: positive sharp wave, SAN: spinal accessory nerve.

**Table 2:** Needle EMG findings of polio survivors.

	Number of muscles (%)				
Muscles examined with Needle EMG	PSW/FP	MUAP amp.↑ dur.↑	Recruitment ↓	Absence of MUAP	At least one abnormality
Trapezius (n = 82)	0 (0)	17 (20.7)	17 (20.7)	1 (12.2)	18 (22.0)
FDI (n = 90)	0 (0)	37 (41.1)	37 (41.1)	2 (2.2)	39 (43.3)
APB (n = 12)	0 (0)	4	4 (33.3)	2 (16.6)	6 (50)
BB (n = 81)	0 (0)	18 (22.2)	18 (22.2)	3 (3.7)	21 (25.9)
Triceps (n = 19)	1 (5.3)	10 (52.6)	11 (57.9)	0 (0)	10 (52.6)
Deltoid (n = 29)	0 (0)	16 (55.2)	16 (55.2)	2 (6.9)	18 (62.1)
TA (n = 138)	6 (4.4)	74 (53.6)	77 (55.8)	38 (27.5)	112 (81.2)
MG (n = 139)	14 (10.1)	42 (30.2)	47 (33.8)	61 (43.9)	103 (93.5)
VL (n = 136)	3 (2.2)	78 (57.4)	80 (58.8)	52 (38.2)	130 (95.6)
PL (n = 19)	0 (0)	9 (47.4)	9 (47.4)	4 (21.1)	13 (68.4)
AM (n = 6)	0 (0)	5 (83.3)	5 (83.3)	0 (0)	5 (83.3)
IP (n = 57)	4 (7)	37 (64.9)	39 (68.4)	8 (14)	45 (79)

AM: adductor magnus, amp.: amplitude, APB: abductor pollicis brevis, BB: biceps brachii, dur.: duration, EMG: electromyography, FDI: first dorsal interosseous, FP: fibrillation potential, IP: iliopsoas, MG: medial gastrocnemius, MUAP: motor unit action potential, n: number, PL: peroneus longus, PSW: positive sharp wave, TA: tibialis anterior, VL: vastus lateralis.

**Table 3:** Needle EMG findings of ALS patients.

	Number of muscles (%)					
Muscles examined with Needle EMG	PSWs / FPs	Fasciculation potentials	MUAP amp.↑ dur.↑	Recruitment ↓	Absence of MUAP	At least one abnormality
Trapezius (n = 23)	14 (60.9)	10 (43.5)	10 (43.5)	15 (65.2)	0 (0)	15 (65.2)
FDI (n = 33)	30 (90.9)	7 (21.2)	10 (30.3)	27 (81.8)	3 (9.1)	30 (90.9)
APB (n = 11)	10 (90.9)	2 (18.2)	2 (18.2)	7 (63.6)	2 (18.2)	10 (90.9)
ADQ (n = 5)	2 (40)	0 (0)	1 (20)	2 (40)	0 (0)	2 (40)
EIP (n = 2)	2 (100)	0 (0)	1 (50)	1 (50)	0 (0)	2 (100)
BB (n = 15)	10 (66.7)	7 (46.7)	9 (60)	0 (0)	0 (0)	10 (66.7)
Deltoid (n = 15)	9 (60)	7 (46.7)	6 (40)	0 (0)	0 (0)	9 (60)
Triceps (n = 6)	4 (66.7)	1 (16.7)	4 (66.7)	0 (0)	0 (0)	4 (66.7)
T11 / T12 (n = 14)	9 (64.3)	5 (35.7)	0 (0)	0 (0)	0 (0)	9 (64.3)
TA (n = 27)	24 (88.9)	2 (7.4)	16 (59.3)	24 (88.9)	1 (3.7)	26 (96.3)
MG (n = 18)	17 (94.4)	2 (11.1)	12 (66.7)	0 (0)	1 (5.6)	17 (94.4)
VL (n = 38)	16 (42.1)	12 (31.6)	19 (50)	0 (0)	0 (0)	21 (55.3)
Tongue (n = 7)	5 (71.4)	5 (71.4)	0 (0)	5 (71.4)	0 (0)	5 (71.4)

amp.: amplitude, ADQ: abductor digiti quinti; APB: abductor pollicis brevis; BB: biceps brachii; dur.: duration; EIP: extensor indicis proprius, EMG: electromyography, FDI: first dorsal interosseous, FP: fibrillation potential, IP: iliopsoas, MG: medial gastrocnemius, MUAP: motor unit action potential, n: number, PSW: positive sharp wave, TA: tibialis anterior, VL: vastus lateralis.

**Table 4:** Demographic and neurophysiological characteristics of patients with SAN lesions.

Patients	Age/Gender	Etiology	Side of the lesion	Timing of the Edx (months)	Latency (ms) Right/left	Amplitude (mV) Right/left	PSWs / FPs	Absent MUAP	MUAP amp.↑ dur.↑
Patient#1	27/M	Mass in the cervical region	R	2	2.0/2.1	8.0/32.4	+	−	−
Patient#2	31/F	Neck dissection	L	24	1.7/Absent	12.5/Absent	−	+	−
Patient#3	46/F	Neck dissection	R	12	3.8/1.6	0.6/10.7	+	−	+
Patient#4	25/F	Neck dissection	L	6	2.0/2.6	13.7/9.3	+	−	+
Patient#5	58/M	Mass in the cervical region (multip. cran. neur.)	R	2	Absent/2.1	Absent/11.5	+	+	−
Patient#6	58/M	Neck dissection	R	2	2.3/1.8	0.2/12.2	+	+	−
Patient#7	26/M	Trauma	L	12	2.7/Absent	20.2/Absent	+	+	−

amp.: amplitude, dur.: duration, Edx: electrodiagnostic test, F: female, FP: fibrillation potential, IP: iliopsoas, L: left, M: male, MG: medial gastrocnemius, MUAP: motor unit action potential, multip. cran. neur.: multiple cranial neuropathies, PSW: positive sharp wave, R: right, SAN: spinal accessory nerve.
